# Persistent pain induces mood problems and memory loss by the involvement of cytokines, growth factors, and supraspinal glial cells

**DOI:** 10.1016/j.bbih.2020.100118

**Published:** 2020-07-25

**Authors:** Morgana D. da Silva, Giselle Guginski, Karina L. Sato, Luciana Sayuri Sanada, Kathleen A. Sluka, Adair R.S. Santos

**Affiliations:** a Laboratory of Neurobiology of Pain and Inflammation, Department of Physiological Sciences, Center for Biological Sciences, Federal University of Santa Catarina, University Campus, Trindade, Florianópolis, SC, 88040-900, Brazil; b Program of Pos-graduation in Neuroscience, Federal University of Santa Catarina, University Campus, Trindade, Florianópolis, SC, 88040-900, Brazil; c Department of Pharmacology, Center of Biological Sciences, Federal University of Santa Catarina, University Campus, Trindade, Florianópolis, SC, 88040-900, Brazil; d Department of Physical Therapy and Rehabilitation Science, Pain Research Program, University of Iowa, #1-252 MEB, Iowa City, IA, 52241, USA

**Keywords:** Neuropathic pain, Mood disorders, Inflammatory cytokines, Neurotrophic factors, Glial activation

## Abstract

Lesions of peripheral nerves lead to pain, hyperalgesia, and psychological comorbidities. However, the relationship between mood disorders and neuropathic pain is unclear, as well as the underlying mechanisms related to these disorders. Therefore, we investigated if nerve injury induces depression, anxiety, and cognitive impairment and if there were changes in cytokines, growth factors, and glial cell activation in cortical sites involved in processing pain and mood in animals with nerve injury. Nerve injury was induced by partial sciatic nerve ligation (PSNL) in male Swiss mice and compared to sham-operated animals. Nociceptive behavioral tests to mechanical and thermal (heat and cold) stimuli confirmed the development of hyperalgesia. We further examined mood disorders and memory behaviors. We show nerve injury induces a decrease in mechanical withdrawal thresholds and thermal latency to heat and cold. We also show that nerve injury causes depressive-like and anxiety-like behaviors as well as impairment in short-term memory in mice. There were increases in proinflammatory cytokines as well as Brain-Derived Neurotrophic Factor (BDNF) in the injured nerve. In the spinal cord, there were increases in both pro and anti-inflammatory cytokines, as well as of BDNF and Nerve Growth Factor (NGF). Further, in our data was a decrease in the density of microglia and astrocytes in the hippocampus and increased microglial density in the prefrontal cortex, areas associated with neuropathic pain conditions.

## Highlights

• Partial sciatic nerve ligation induces hypersensitivity in mice.
• Nerve injury induces depressant-like and anxiolytic-like behavior and memory loss.
• The nerve and spinal cord showed an increase in cytokines and neurotrophins.
• Nerve injury decreases density of microglia and astrocytes in the hippocampus.
• Also, injury increases microglial density in the prefrontal cortex.

## Graphical abstract

**Figure:**
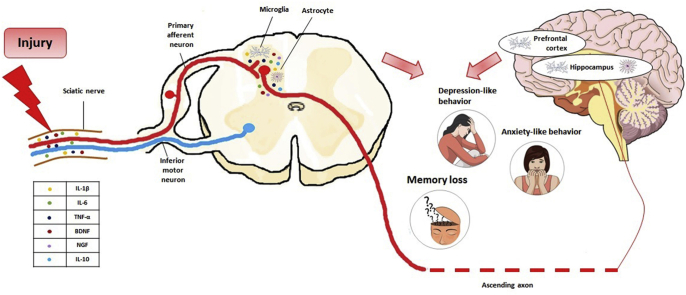

## Teaser

Supraspinal astrocytes and microglia are involved in the pathogenesis of PSNL-induced painful neuropathy and mood disorders, as well as the increase of inflammatory factors in the spinal cord and nerve. This measure could potentially help clinicians to develop better treatment for chronic pain problems.

## 1 Introduction

Neuropathic pain is caused by a lesion or disease of the somatosensory nervous system by direct insult, illness, or drug toxicity - associated with no-typical sensory changes including pain that occurs without any other previous stimulation as well as altered sensory perception (hyperalgesia and allodynia) (Malmberg and Basbaum, 1998; Jensen et al., 2011). Neuropathic pain affects almost 8% of the general population, it is difficult to treat, and it significantly impacts the quality of human life (Szok et al., 2019). Additionally, neuropathic pain is associated with psychological comorbidities such as depression, anxiety (Arora and Chopra, 2013; Blackburn-Munro and Blackburn-Munro, 2001; Rouwette et al., 2012) and cognitive impairment (Moriarty et al., 2011). The psychological comorbidities have shared neurobiology with that of nociception including central neuroanatomical and neurochemistry pathways, like as noradrenaline and serotonin (Manji et al., 2001).

Neuroinflammation in the spinal cord is involved in nerve injury models of chronic pain. Growth factors and inflammatory cytokines, produced by spinal microglia and astrocytes, contribute to enhanced excitability of dorsal horn neurons and subsequently contribute to persistent pain (Biggs et al., 2010; Latremoliere and Woolf, 2009; Walters, 2014). While supraspinal sites, both brainstem and cortical, have been implicated in nociceptive processing including nerve injury (Jaggi and Singh, 2011; Walters, 2014; Clark et al., 2013), it is unclear if nerve injury alters glial cell activity outside the spinal cord, particularly in cortical sites that process nociceptive information. Cortical sites involved in nociceptive processing include the somatosensory cortices (primary and secondary) which mediates the sensation of pain, the anterior insular and anterior cingulate cortices that are related to the emotional component of pain, the prefrontal cortex (PFC) which is involved in complex cognitive behaviors related to pain and the motor cortices involved with movement responses to pain (Peltz et al., 2011; Sanada et al., 2014).

Glial cells also contribute to the pathophysiology of mood disorders. In individuals with major depressive disorder, there is a decrease in the number of cells in multiple cortical regions including prefrontal and cingulate cortices (Cotter et al., 2001, 2002), and there is a reduced volume of the hippocampus (Baumann et al., 1999) and PFC (Drevets, 2000). Further increased pro-inflammatory cytokines (Dahl et al., 2014) and reduction of neurotrophins (Biggs et al., 2010; Peltz et al., 2011) in the hippocampus are related to mood disorders as well as synaptic connections in the PFC and hippocampus are decreased by chronic stress exposure, which reduces the local expression of BDNF in animals (Duman and Monteggia, 2006).

The purpose of the present study was to investigate if nerve injury-induced not only alterations in nociceptive behaviors but also co-morbid symptoms of depression, anxiety, and cognitive impairment, and if there were alterations in glia and glial-derived neuromodulators in cortical sites typically involved in processing nociceptive stimuli and mood. We, therefore, examined i) nociceptive behaviors, depression, anxiety, and memory after nerve injury, ii) growth factors and cytokines levels in peripheral nerve, spinal cord or central areas, and iii) glial cell activation in cortical sites.

## 2 Materials and methods

### 2.1 Animals

Male Swiss mice (30–40 ​g) were acquired from the Federal University of Santa Catarina (UFSC)/Brazil and the University of Iowa (Iowa City, IA, USA). All the methods were approved by the Institutional Ethics Committee (CEUA/UFSC, protocol number PP00208 and PP00745, and Iowa protocol number 0908193). They were housed with a 12 ​h light-dark cycle (lights on at 07:00 a.m.) and received food and water *ad libitum*. We work according to the "Principles of Laboratory Animal Care" from the National Institutes of Health. Animals were acclimatized to the research laboratory for at least 1 ​h before behavioral tests. All animals were tested for hypersensitivity before behavioral assessments of depression, anxiety, and memory. We used 32 animals - 8 for each behavioral evaluation, and the samples of these same mice were used for the analysis of cytokines and neurotrophins. Another 10 animals were used to perform the analysis of glial cells, totaling 42 animals in the current work.

### 2.2 Partial sciatic nerve ligation (PSNL)

Mice were anesthetized with 2% isoflurane via a nose cone for 5 ​min (a total maximum time). An incision was made at the trochanter level of the femur, and the muscles surrounding the nerve were separated. A PSNL was performed by tying the distal 1/3 to 1/2 of the dorsal portion of the right sciatic nerve, according to the procedure described in mice (Malmberg and Basbaum, 1998). In sham-operated mice, the sciatic nerve was exposed without ligation. The wound was closed, and animals allowed to recover for 7 days before starting the tests.

### 2.3 Behavioral assessments

**Fig. 1:**
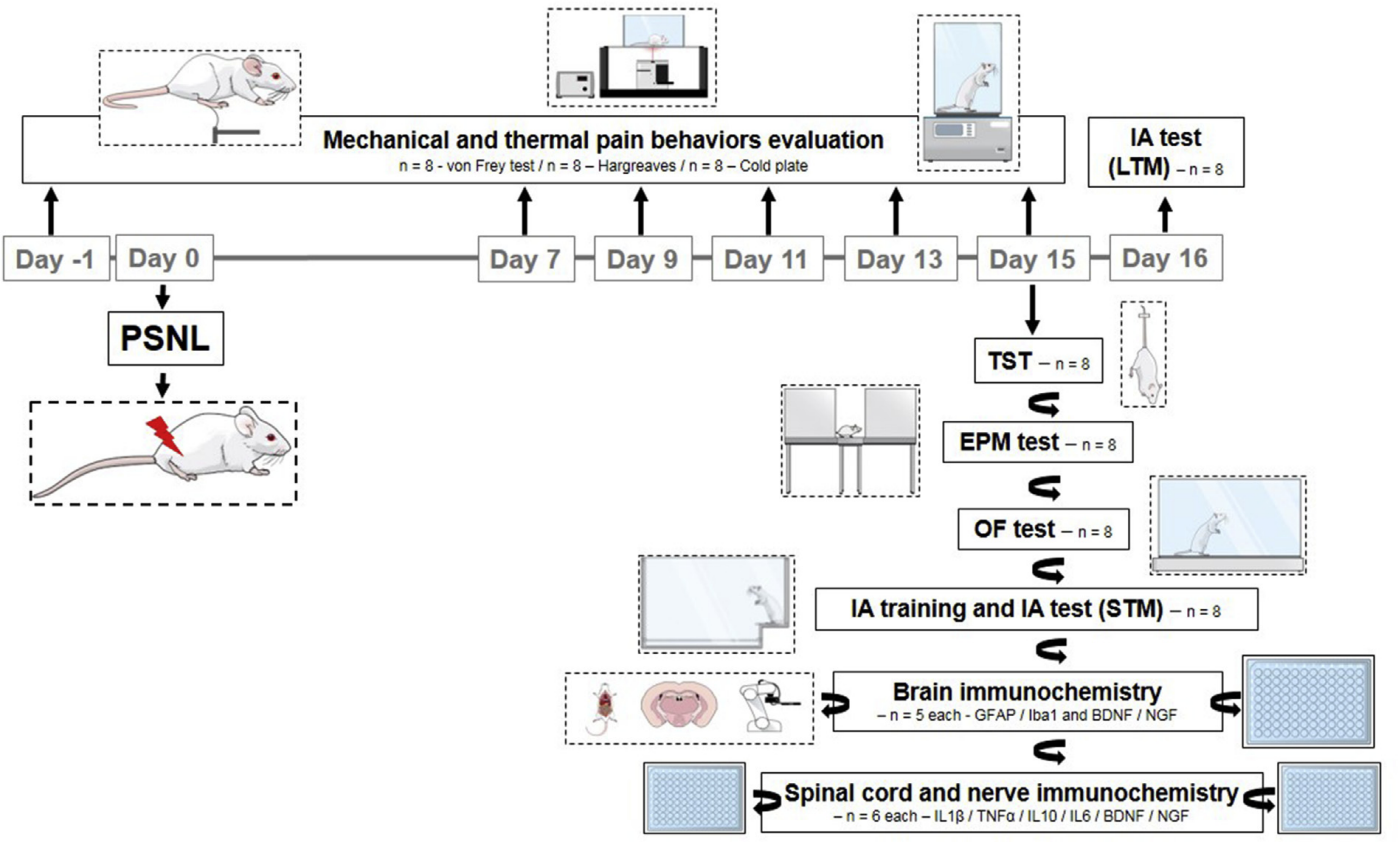
Experimental design. The surgery (PSNL) was realized on Day 0. Mechanical and thermal pain behaviors were evaluated in 7, 9, 11, 13, and 15 days (grey) after surgery. The mood disorders behavioral and memory assessments were conducted on Day 15 and 16. A different group of animals was used to perform the hippocampus and cerebral cortex glial cells analysis (n ​= ​5).

The experimental design is shown in Fig. 1. These behavioral tests examined nociceptive, depressive-like, anxiety-like, and memory-related behaviors. A series of behavioral experiments were observed in animals before and after PSNL: Mechanical sensitivity, Thermal sensitivity to heat, and cold. Depressive and anxiolytic behaviors were examined fifteen days after surgery. Memory behaviors were observed fifteen and sixteen days after surgery. Detailed procedures for each are outlined below.

#### 2.3.1 Mechanical sensitivity induced by partial sciatic nerve ligation (PSNL)

We used von Frey filaments (Stoelting, Chicago, USA) (Chaplan et al., 1994) and determined mechanical sensitivity according to the up-and-down method (Dixon, 1980). It was calculated by the force which elicited a 50% paw withdrawal threshold in response to the application of different filaments (0.02–4 ​g initiated with the 0.4-g filament and effect determined in grams). We placed each mouse in clear plexiglass boxes (9 ​cm ​× ​7 ​cm x 11 ​cm) on an elevated wire mesh platform accessing the ventral surface of the right hind paw. The filaments were applied and sustained about 3 ​s from below the grid floor perpendicular to the plantar surface. If the paw was withdrawn next weaker filament was applied and the subsequent measurement recorded, if the animal did not respond, the following more robust thread was used and continued until six responses. These sequence responses were used to interpolate the threshold. The frequency of withdrawal was determined before nerve injury (baseline) and compared to 7, 9, 11, 13, and 15 days after PSNL.

#### 2.3.2 Thermal sensitivity to heat-induced by partial sciatic nerve ligation (PSNL)

Thermal sensitivity was tested with the Hargreaves test (Hargreaves et al., 1988). The animals were placed in acrylic cubicles on a glass surface. Infrared light (40 ​°C) was shown on the hind paws, and the time for the animal to remove its right hind paw from heat source were recorded automatically. Three trials, with 5–10 ​min in between, were taken at each time point and averaged. A cut off time of 12 ​s was used. Thermal sensitivity to heat was evaluated before nerve injury (baseline) and compared to 7, 9, 11, 13, and 15 days after injury.

#### 2.3.3 Thermal sensitivity to cold-induced by partial sciatic nerve ligation (PLSN)

Thermal sensitivity to cold was tested with a cold plate (HOT-COLD CASE MOD, AVS CQF, São Paulo, Brazil) as previously described (Bennett and Xie, 1988) with modifications. The animals were acclimated on the surface of the plate at room temperature (22 ​± ​2 ​°C) for 5 ​min/day, two days before the start of the evaluations. On the day of testing, mice were placed individually on the cold plate (10 ​± ​1 ​°C) in a glass container (25 ​× ​30 ​× ​30 ​cm). The latency to withdrawal determined by shaking or licking of the right hind paw was considered a positive response. A cut off time of 2 ​min was used to avoid tissue damage. Each temperature was tested in all mice with 1 ​h between tests. All groups were evaluated with the same procedures above before and 7, 9, 11, 13, and 15 days after the surgical procedures.

#### 2.3.4 Tail suspension test (TST)

We used the method described previously by Steru et al. (1985). Mice were deprived of visual and acoustic stimuli remaining isolated in the test room. To carry out the test, they were suspended 50 ​cm above the floor by adhesive tape placed 1 ​cm from the tip of the tail, and a video camera was placed in front of the animal to record their behavior for 6 ​min. Immobility time was manually recorded by a trained observer who received the videos without knowing which group was the animal (blind evaluator). Mice were considered immobile only when they hung passively and wholly motionless, and the total duration of immobility induced by tail suspension was measured. All groups were evaluated 15 days after nerve PSNL.

#### 2.3.5 Inhibitory avoidance task (IA)

The IA apparatus was a 50 ​× ​25 ​× ​25-cm acrylic box, whose floor consisted of parallel 1.0 ​mm diameter stainless steel bars spaced 1.0 ​cm apart. A 10-cm2, 2-cm high, platform occupied the center or the floor. In the training session, immediately after stepping down and placing their four paws on the grid, animals received a 0.4 ​mA, 2.0 ​s scrambled foot shock. In test sessions, no foot shock was used. Step-down latency (with a maximum of 180 ​s) was used as a measure of aversive memory retention. Animals were tested 1 ​h (Short-term memory-STM) and 24 ​h (long-term memory-LTM) after training (Barros et al., 2006). All groups were evaluated 15 (STM) and 16 (LTM) days after nerve injury.

#### 2.3.6 Open field test (OF)

Ambulatory behavior was assessed in an OF, as described previously (Rodrigues et al., 1996). The apparatus consisted of a wooden box measuring 40 ​× ​60 ​× ​50 ​cm. The floor of the arena was divided into 12 equal squares. The central squares are regarded as the "center" of the field. A 60 ​W light bulb was positioned approximately 1 ​m above the arena. Mice were transferred 1 ​h beforehand to the testing room and placed in the center of the field. The following parameters were manually scored: the number of squares crossed (defined as at least three paws in a square), the number of times an animal reared, and the time spent in the center of the arena during a 6-min test. Less time spent in the central area is usually taken as a measure of a higher level of anxiety. After each inspection, the arena was sprayed with 70% ethanol and wiped thoroughly to remove the residual odor (Bahi et al., 2014). All groups were evaluated 15 days after the nerve injury.

#### 2.3.7 Elevated plus-maze test (EPM)

The EPM apparatus consisted of a central platform (10 ​× ​10 ​cm) with four arms (45 ​× ​10 ​cm), of which two were open, and two were closed. The open arms are arranged to face each other, and the closed arms also arranged in front of each other, forming a kind of cross. The apparatus has a pedestal keeping 88 ​cm above floor level. Each mouse was placed on the central platform of the plus-maze facing an open arm. Cameras of videos were placed on top of the platform and recorded by 5 ​min. Time spent in the open and enclosed arms and the number of entries were manually analyzed by a trained observer who received the recording without knowing which group was the animal (blind evaluator). The percentage of time spent in the enclosed arms and the number of entries in these arms were used as a measure of anxiety (Pellow et al., 1985). All groups were evaluated 15 days after the induction of nerve injury.

### 2.4 Determination of cytokines levels in the spinal cord and sciatic nerve

Fifteen days after PSNL, we removed the sciatic nerve and the lumbar spinal cord (L1–L6). The tissue was then frozen at −80 ​°C until assayed. Tissues were homogenized in buffer containing Tween 20, benzethonium chloride, ethylenediaminetetraacetic acid -EDTA, ýBovine serum albumin –BSA, NaCl, Phenylmethanesulfonyl fluoride, and aprotinin. The supernatants were processed for IL-1β, TNF-α, IL-10, and IL-6 using enzyme-linked immunosorbent assay (ELISA) from R&D Systems (Minneapolis, MN). Sample aliquots of 100 ​μl were used to measure all cytokines using mouse ELISA kits according to the manufacturer’s instructions. Levels of IL-1β, TNF-α, IL-10, and IL-6 were estimated by interpolation from a standard curve by colorimetric measurements at 450 ​nm (correction wavelength 540 ​nm) in an ELISA plate reader (Berthold Technologies – Apollo 8 – LB 912, KG, Germany). The total protein content of the supernatant was measured using the Bradford method (Schleicher and Wieland, 1978). Results obtained with the use of the ELISA kits in the analysis of cytokines and growth factors strongly indicate it can be used for quantifying proteins in tissue samples (Bobinski et al., 2018).

### 2.5 Determination of Brain-Derived Neurotrophic Factor (BDNF) and Nerve Growth Factor (NGF) levels

Fifteen days after PSNL, we isolated the sciatic nerve, lumbar spinal cord (L1–L6), hippocampus, and the cerebral cortex. The tissue was then frozen at −80 ​°C until assayed. The same procedures described above were performed (homogenization of the tissue, amount of sample, and stipulation of the data). The supernatants were processed for BDNF and NGF using enzyme-linked immunosorbent assay (ELISA) from R&D Systems (Minneapolis, MN) according to the manufacturer’s instructions and the total protein content of the supernatant was measured using the Bradford method.

### 2.6 Brain microglia and astrocytes analysis

Fifteen days after PSNL mice were deeply anesthetized with 25% urethane (1 ml/100 ​g) and transcardially perfused through the left ventricle with PBS 0.05 ​M followed by 4% phosphate-buffered paraformaldehyde. The brain was then removed, postfixed in 4% paraformaldehyde for 4h before transferring to 30% sucrose solution for 48h. Sections were then frozen on dry ice and stored at −80 ​°C until sectioned. Coronal sections (20 ​μm) of the brain were cut at a cryostat and placed on slides in serial order, including hippocampus areas (CA1, CA2, CA3, CA4, dentate gyrus – DG) and cerebral cortex areas: a) Cingulate; b) Insula; c) motor - M1; d) pre-motor - M2; e) Pre-frontal; f) primary somatosensory cortex - S1 HL; and g) secondary somatosensory cortex - S2 (Pathak et al., 2014). For immunohistochemistry, 10 brains were chosen (5 in each group from PSNL and sham groups).

Microglial cells were examined by immunohistochemical staining the tissue with Iba1. Iba1 is a 17-kDa EF-hand protein that is specifically expressed in microglia/macrophage and is upregulated during the activation of these cells. The sections were first blocked with 3% normal goat serum for 30 ​min, followed by Avidin-Biotin Block (15 ​min each). The article was then incubated overnight with mouse anti-rabbit Iba1 (AbDSerotec, Raleigh, NC, USA—1:2500, Cat. no. MCA275G). On the second day, sections were incubated in biotinylated goat anti-mouse IgG (Invitrogen, Carlsbad, CA, USA—1:1000) for 1h, followed by incubation in Strep-568 (Invitrogen, Carlsbad, CA, USA—1:1000) for 1 ​h. Slides were coverslipped using Vectashield (Vector Laboratories, Burlingame, CA, USA).

The second set of slides were double-labeled with anti-GFAP (Glial Fibrillary Acidic Protein) and anti-MCP-1 (Monocyte Chemotactic Protein-1) to visualize astrocytes. GFAP is one of a family of intermediate filament proteins essential for the process of reactive astrogliosis, and MCP-1 is a neuroprotective chemokine. The slides were first blocked with 3% normal goat serum for 30 ​min, followed by Avidin-Biotin Block (15 ​min each). Then, the sections were incubated overnight with a monoclonal anti-mouse anti-GFAP (Millipore, Billerica, MA, USA—1:5000, Cat. no. MAB360). On the second day, the sections were incubated with biotinylated goat anti-mouse IgG (Invitrogen, Carlsbad, CA, USA—1:1000) for 1 ​h, followed by Strep-568 (Invitrogen, Carlsbad, CA, USA—1:1000) for 1 ​h. Slides were then incubated overnight in rabbit anti-rat MCP-1 (Millipore, Billerica, MA, USA—1:500, Cat. no. 1834P). Section was then incubated with biotinylated goat anti-rabbit IgG (Invitrogen, Carlsbad, CA, USA—1:1000) for 1 ​h, followed by Strep-488 (Invitrogen, Carlsbad, CA, USA—1:1000) for 1 ​h. Slides were covered using Vectashield (Vector Laboratories, Burlingame, CA, USA). All sections were stained simultaneously to avoid differences between the days of testing.

The researcher who took and analyzed the photos was blind, unable to identify the experimental groups. Images were captured using a fluorescence microscope (Olympus BX-51—Japan) and analyzed off-line. All photos were taken with the same settings, from the same brain area for comparison. Density was determined using ImageJ software as previously described by us (Hoeger-Bement and Sluka, 2003). Five sections per area of the brain (randomized every 3 cuts) for each mouse were averaged for each strain. The number of pixels was determined. To analyze the picture, each tissue section was open in ImageJ and converted to an eight-bit grayscale. Images were inverted, and each tissue section was calibrated independently using the "uncalibrated OD" function with pixel values ranging from 0 to 255 to prove a density value as pixels per area.

### 2.7 Statistical analysis

We confirmed the normality using the Shapiro-Wilk analysis. The results were evaluated statistically using a post-hoc test Bonferroni, two-way analysis of variance (ANOVA) (Mechanical and thermal sensitivity evaluations), and nonparametric test (Unpaired *t*-test) (behavioral assessments, neurotrophins, and cytokines levels and glial analysis). We used *GraphPad* Software (San Diego, CA, USA) to develop graphics and statistical analysis. The results are presented as means ​± ​standard error of the mean (SEM), which are reported as geometric means accompanied by their respective 95% confidence limits, and the significance level in all cases was set at P ​< ​0.05.

## 3 Results

### 3.1 Nociceptive behaviors in neuropathic mice

**Fig. 2:**
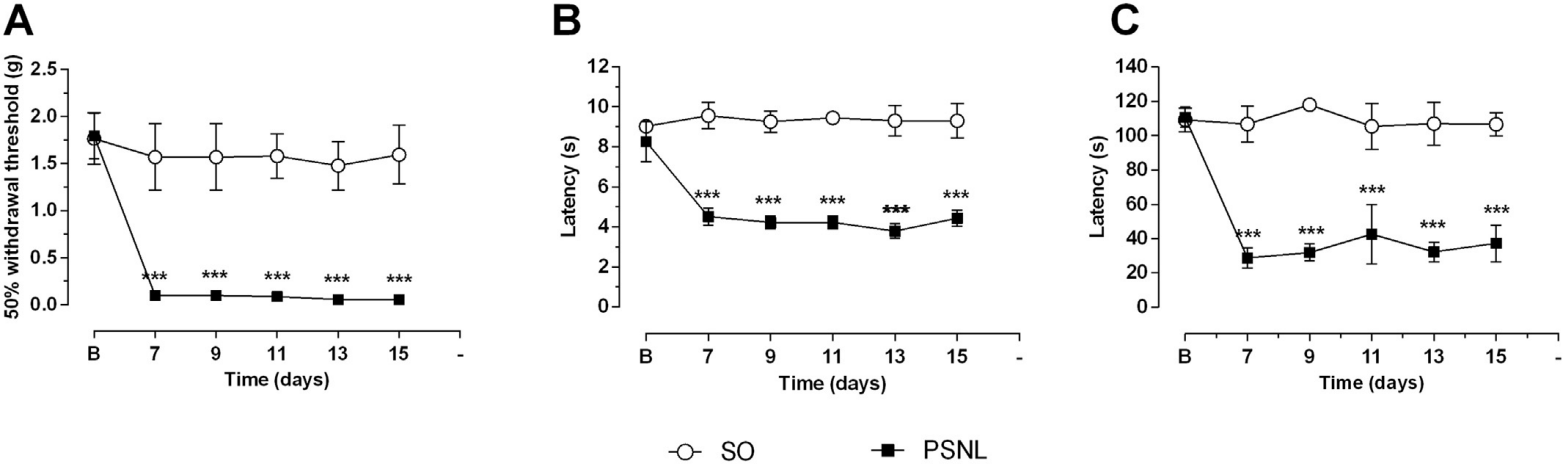
Effect of PSNL on mechanical and thermal pain behaviors. Animals were randomly divided into two groups: Sham-operated (SO) and operated (PSNL). (A) Mechanical sensitivity evaluated by von Frey Hair filaments in sham-operated (SO) and operated (PSNL) animals (n ​= ​8). PSNL animals withdrew the paw to the stimulus of more delicate von Frey filaments. (B) Thermal sensitivity evaluated by the Hargreaves test in sham-operated and operated (PSNL) animals (n ​= ​8). (C) Thermal sensitivity assessed by the cold plate at 10 ​°C in sham-operated and operated (OP) animals (n ​= ​8). Letter B corresponds to evaluation before surgical procedure. Seven days after PSNL returned the assessments (7, 9, 11, 13, and 15 days after surgery). (■) represents the PSNL group (PSNL); (○) represents the sham-operated group (SO). The vertical lines indicate the S.E.M, and the symbols indicate: ∗∗∗P ​< ​0.001 denote significance levels when compared with the sham-operated group (2 way ANOVA followed by Bonferroni post hoc test).

Nerve injury-induced a significant decrease in paw withdrawal threshold when compared to values before surgery or sham-operated mice (Fig. 2A). PSNL induced decreases in withdrawal threshold were significant (P ​< ​0.001) on days 7 (PSNL mean ​= ​0.097 ​g versus SO mean ​= ​1.569 ​s), 9 (PSNL mean ​= ​0.097 ​g versus SO mean ​= ​1.569 ​s), 11 (PSNL mean ​= ​0.087 ​g versus SO mean ​= ​1.579 ​s), 13 (PSNL mean ​= ​0.056 ​g versus SO mean ​= ​1.477 ​s) and 15 (PSNL mean ​= ​0.055 ​g versus SO mean ​= ​1.594 ​s) when compared to the sham-operated group (Fig. 2A).

Nerve injury-induced a significant decrease in thermal withdrawal latency to noxious heat stimuli when compared to values before surgery or sham-operated animals (Fig. 2B). PSNL induced decrease in heat withdrawal latency were significant on days 7 (PSNL mean ​= ​4.516 ​s versus SO mean ​= ​9.566 ​s), 9 (PSNL mean ​= ​4.23 ​s versus SO mean ​= ​9.266 ​s), 11 (PSNL mean ​= ​4.23 ​s versus SO mean ​= ​9.450 ​s), 13 (PSNL mean ​= ​3.8 ​s versus SO mean ​= ​9.303 ​s) and 15 (PSNL mean ​= ​4.43 ​s versus SO mean ​= ​9.308 ​s) when compared to the sham-operated group (P ​< ​0.001) (Fig. 2B).

PSNL induced a significant decrease in paw sensitivity to cold (P ​< ​0.001) when compared to the SO group (Fig. 2C). Nerve injury-induced decreases in cold withdrawal threshold on days 7 (PSNL mean ​= ​28.68s), 9 (PSNL mean ​= ​31.89s), 11 (PSNL mean ​= ​42.57s), 13 (PSNL mean ​= ​32.16s) and 15 (PSNL mean ​= ​37.21s) when compared to the sham-operated group. PSNL reduced paw withdrawal to a minimum value of 28.68s (day 7, P ​< ​0.001).

### 3.2 Mood behaviors in neuropathic mice

**Fig. 3:**
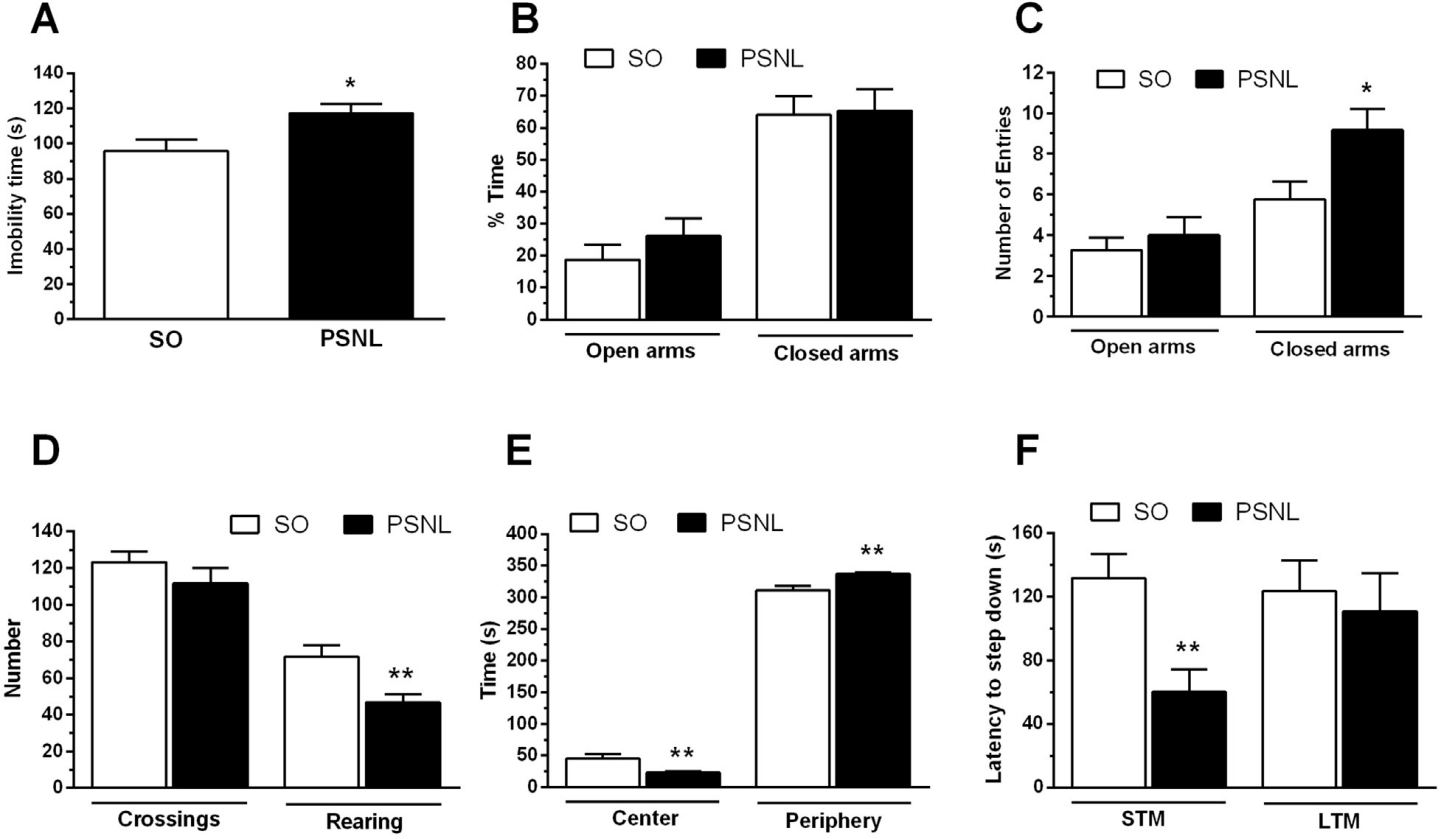
Effect of PSNL on a predictive animal model for depression (TST), on memory (IA test), on elevated plus maze (EPM) and open field (OF) tests in mice. Animals were randomly divided into two groups: Sham-operated (SO) and operated (OP). (A) Depressant-like behavior evaluated by the Tail suspension test (TST) in sham-operated (SO) and operated (PSNL) animals (n ​= ​8). (B) Short-term memory (STM) and long-term memory (LTM) evaluated by Inhibitory Avoidance Task (IA) in sham-operated and operated (OP) animals (n ​= ​8). (C and D) Anxiolytic-like behavior evaluated by Elevated plus-maze test (EPM) in sham-operated (SO) and operated (PSNL) animals (n ​= ​8). C shows the percentage of time spend by animals in open and closed arms, and D indicates the number of entries in open and closed arms of EPM. (E and F) Anxiolytic-like behavior evaluated by Open Field test (OF) in sham-operated (SO) and operated (PSNL) animals (n ​= ​8). E shows the time spent by animals in the center or on the periphery of the OF apparatus. F shows an animal’s number of crossing and number of rearing in OF equipment. All animals were analyzed fifteen days after sham or PSNL surgery. The vertical lines indicate the S.E.M and the symbols indicate: ∗P ​< ​0.05 and ∗∗P ​< ​0.01 denote significance levels when compared with the sham-operated group (Unpaired *t*-test).

To test for depressive-like symptoms, we examined mice for immobility time using the tail suspension test. An increase in tail suspension time occurred in PSNL mice on Day 15 when compared to the sham-operated mice (P ​< ​0.05), indicating that the PSNL induces a depressive-like behavioral (mean of SO ​= ​95.56 ​s and PSNL ​= ​117.11 ​s) (Fig. 3A).

To test for anxiety-like symptoms, we examined the time remained in the open and closed arms and the number of times the mouse entered each division of the elevated plus-maze test. There were no differences in the amount of time spent in the open or closed arms (Fig. 3B), nor in the number of times the mice entered the open arms in sham-operated and PSNL group (Fig. 3C). However, the number of times the mouse came the closed arms - more as a measure of motor activity - was significantly higher in PSNL animals (P ​< ​0.05 – SO ​= ​5.75 and PSNL ​= ​9.17 number of entries) (Fig. 3D).

We also evaluated anxiety-like symptoms for preference time in central or peripheral areas, rearing, and the number of line crossings using the open-field test (OF). There were no significant differences between groups for the number of passages (Fig. 3D). However, the number of times the animal reared was significantly decreased in the PSNL group when compared to the sham group (P ​< ​0.01 – SO ​= ​71.71 ​s and PSNL ​= ​46.62 ​s) (Fig. 3D). Further, the time spent in the center of the open-field was lower (mean SO ​= ​45 ​s and PSNL ​= ​23 ​s) and the time spent in the periphery of the open field OF was higher (mean SO ​= ​311.17 ​s and PSNL ​= ​337 ​s) in PSNL mice when compared to sham mice (P ​< ​0.01) (Fig. 3E).

To test mice for memory deficits, we examined animals for step-down latency using the inhibitory avoidance task (IA). A decreased for short-term memory (STM) (P ​< ​0.01), but not in long-term memory (LTM), occurred in neuropathic injured mice fifteen days after surgery when compared to the sham-operated mice (Fig. 3F). The mean of step-down latency in the test was significantly lower in the PSNL group (60.19 ​s) than the sham-operated group (131.57 ​s).

### 3.3 Cytokines, BDNF and NGF levels and immunochemistry in nerve and spinal cord after PSNL

**Fig. 4:**
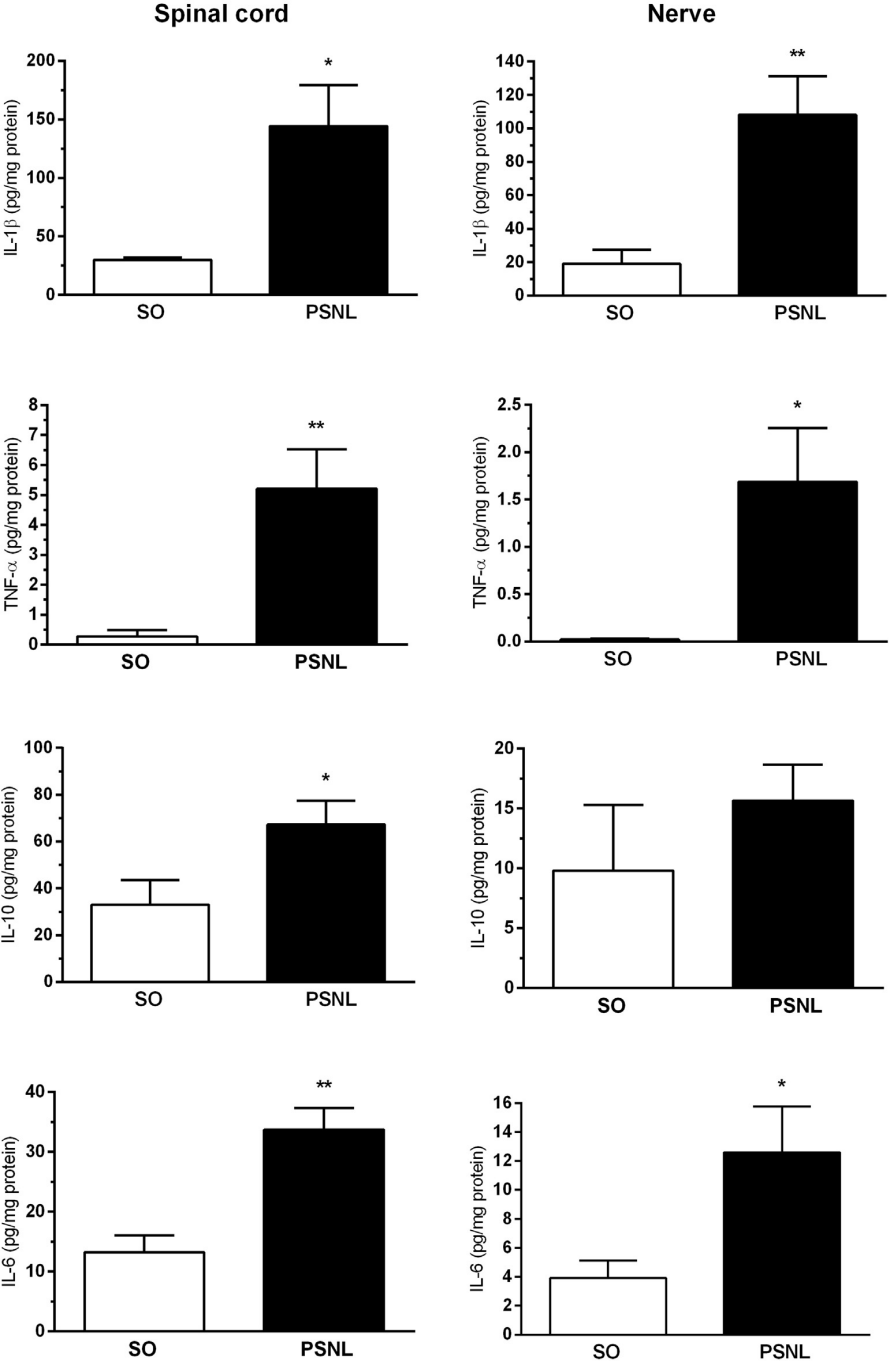
Effect of PSNL on IL-1β, TNF-α, IL-10, and IL-6 levels in the spinal cord and sciatic nerve. Animals were randomly divided into two groups: Sham-operated (SO) and operated (OP). (A and B) Amount of IL-1β in spinal cord and sciatic nerve in sham-operated (SO) and operated (PSNL) animals (n ​= ​6). (C and D) Amount of TNF-α in the spinal cord and sciatic nerve in sham-operated (SO) and operated (PSNL) animals (n ​= ​6). (E and F) Amount of IL-10 in the spinal cord and sciatic nerve in sham-operated (SO) and operated (OP) animals (n ​= ​6). (G and H) Amount of IL-6 in the spinal cord and sciatic nerve in sham-operated (SO) and operated (PSNL) animals (n ​= ​6). The spinal cord and nerve were removed fifteen days after sham or PSNL surgery. The vertical lines indicate the S.E.M and the symbols indicate: ∗P ​< ​0.05 and ∗∗P ​< ​0.01 denote significance levels when compared with the sham-operated group (Unpaired *t*-test).

**Fig. 5:**
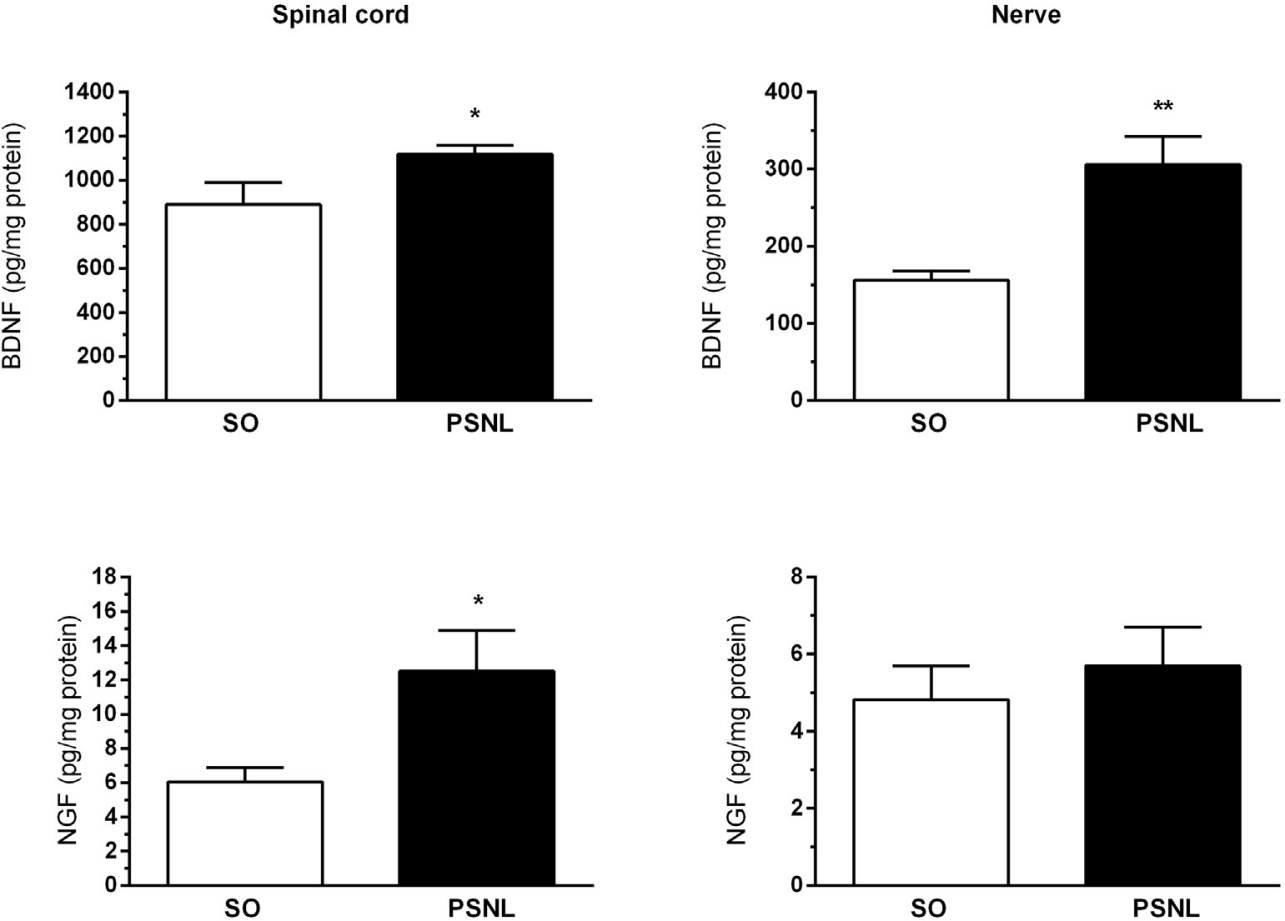
Effect of PSNL on BDNF and NGF levels in the spinal cord and sciatic nerve. Animals were randomly divided into two groups: Sham-operated (SO) and operated (PSNL). (A and B) Amount of BDNF in the spinal cord and sciatic nerve in sham-operated (SO) and operated (PSNL) animals (n ​= ​6). (C and D) Amount of NGF in the spinal cord and sciatic nerve in sham-operated (SO) and operated (PSNL) animals (n ​= ​6). The spinal cord and sciatic nerve were removed fifteen days after sham or PSNL surgery. The vertical lines indicate the S.E.M and the symbols indicate: ∗P ​< ​0.05 and ∗∗P ​< ​0.01 denote significance levels when compared with the sham-operated group (Unpaired *t*-test).

To assess the number of cytokines and neurotrophins in the central and peripheral nervous system in response to neuropathy, we performed ELISA of the lumbar spinal cord and sciatic nerve after PSNL (operated group) compared to the spinal cord and sciatic nerve without lesion (SO group) in Swiss mice (Fig. 4, Fig. 5). Interestingly, levels of all substances analyzed - IL-1β, TNF-α, IL-10, IL-6, BDNF, and NGF - were significantly increased in the spinal cord when compared to the sham (SO) group (Fig. 4, Fig. 5A, C), with P ​< ​0.05 to IL-1β, IL10, BDNF and NGF, and P ​< ​0.01 to TNF-α and IL6. In the sciatic nerve levels of IL-1β, TNF-α, IL-6 and BDNF were increased when compared to the sham (SO) group (Fig. 4, Fig. 5B), with P ​< ​0.05 to TNF-α and IL6, and P ​< ​0.01 to IL-1β and BDNF.

### 3.4 Brain immunochemistry

**Fig. 6:**
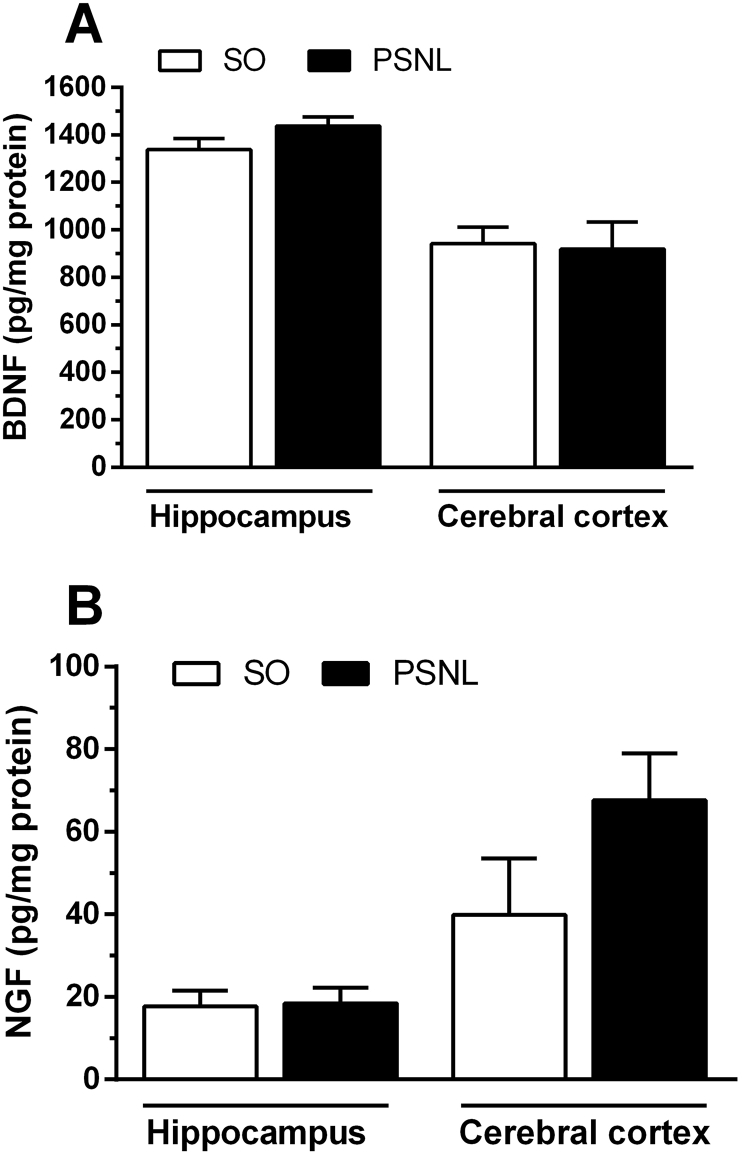
Effect of PSNL on BDNF and NGF levels in the hippocampus and cerebral cortex. Animals were randomly divided into two groups: Sham-operated (SO) and operated (PSNL). (A) Amount of BDNF in the hippocampus and cerebral cortex in sham-operated (SO) and operated (PSNL) animals (n ​= ​6). (B) Amount of NGF in the hippocampus and cerebral cortex in sham-operated (SO) and operated (PSNL) animals (n ​= ​6). The vertical lines indicate the S.E.M.

To assess the amount of neurotrophins in the central nervous system fifteen days after PSNL, we analyzed the levels of BDNF and NGF in the hippocampus and cerebral cortex of mice. There were no differences in the levels of these growth factors between the sham-operated animals and the PSNL (Fig. 6A and B).

**Fig. 7:**
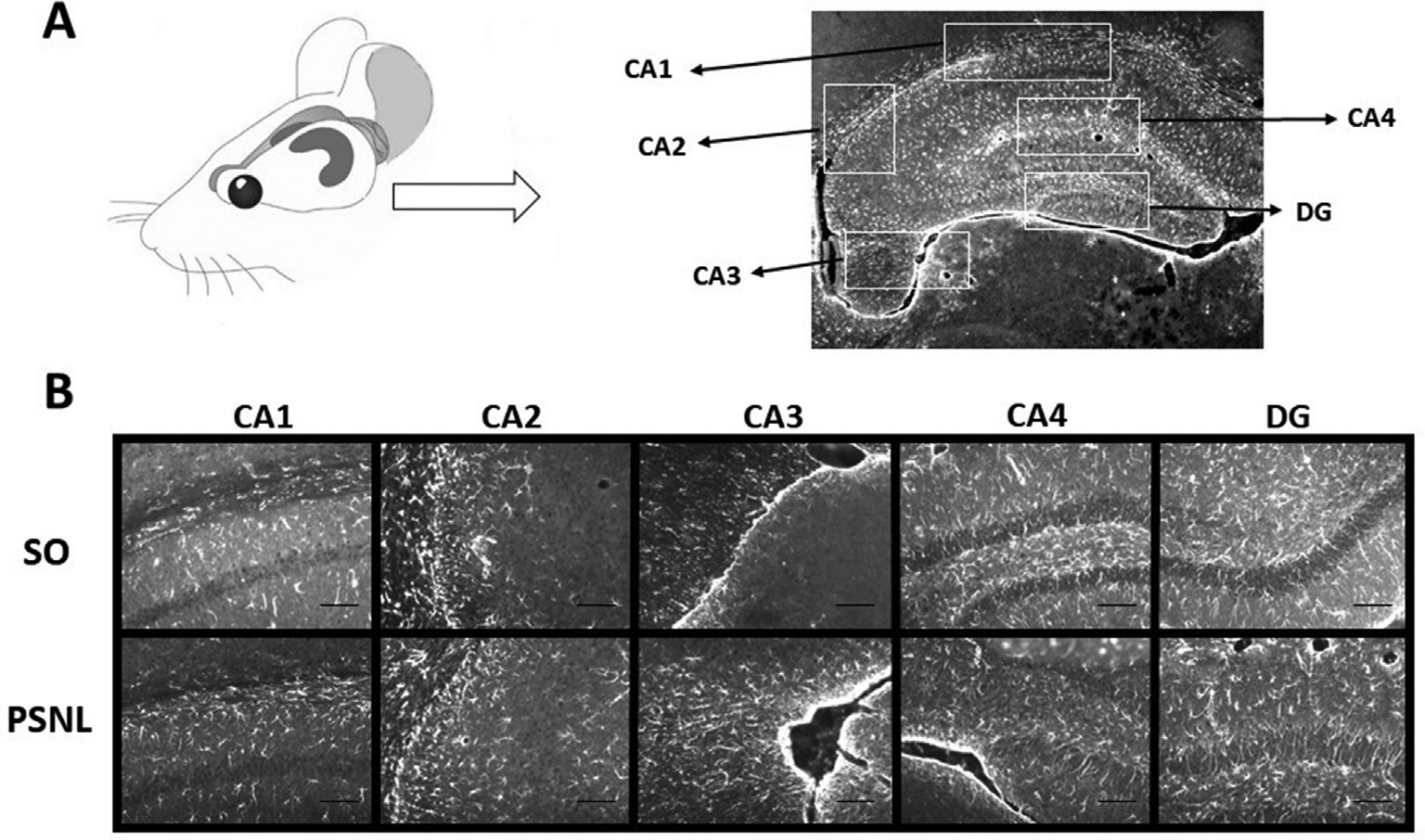
Representative hippocampus-sections immunostained for Iba1. (A) Representative figure of the hippocampus analyzed areas (4X). (B) Representative figures of each hippocampus analyzed areas – CA1, CA2, CA3, CA4, and DG - separately (20X). Groups: SO (sham-operated), PSNL (operated).

**Fig. 8:**
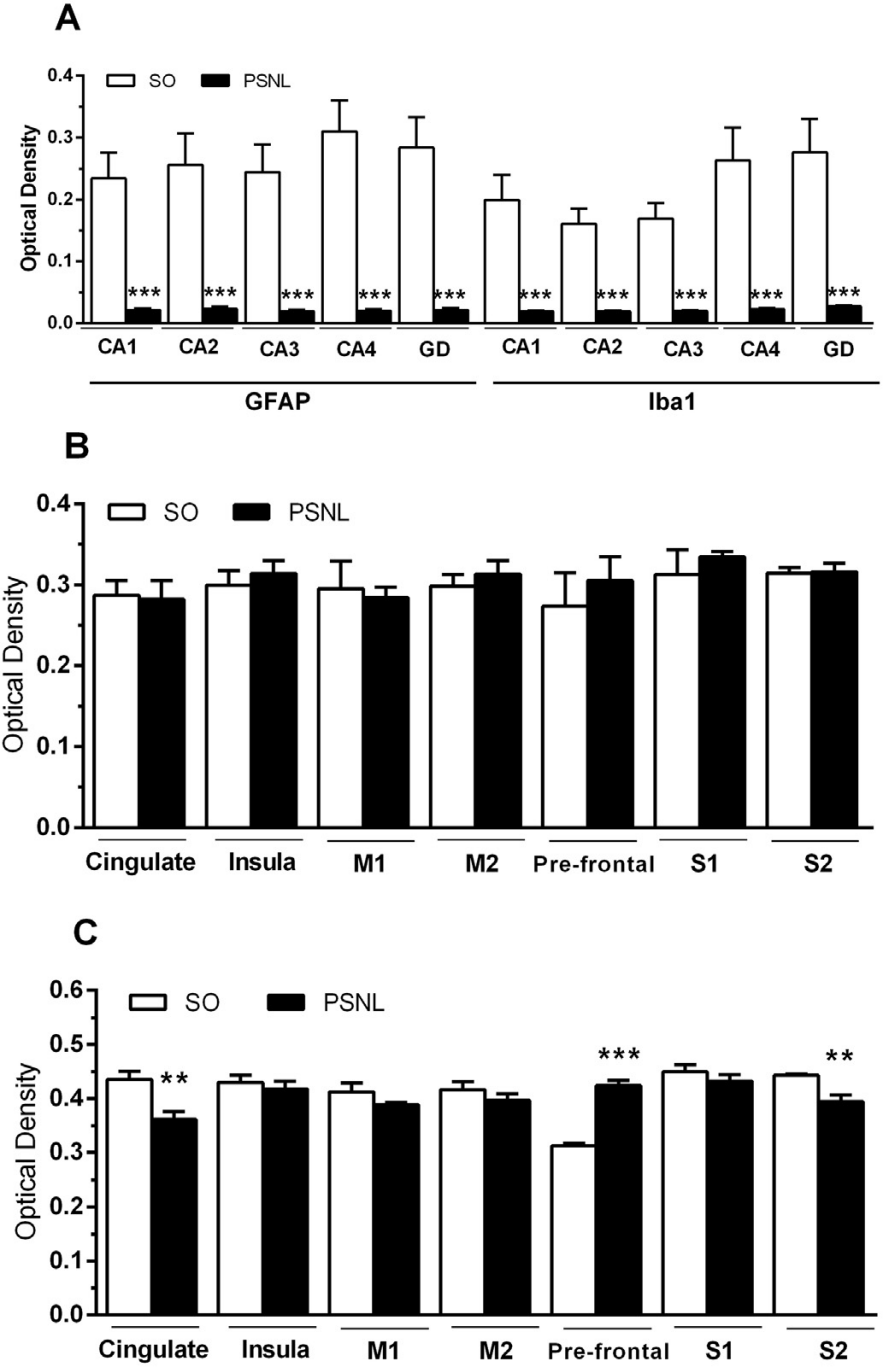
Effect of PSNL in the hippocampus and cerebral cortex glial cells in mice. Average density values of GFAP and Iba1 immunostaining in mice hippocampus and cortex areas. Animals were randomly divided into two groups: SO (sham-operated) and PSNL (operated). (A) Average density values of GFAP and Iba1 immunostaining in the hippocampus (CA1, CA2, CA3, CA4, and DG). (B) Average density values of Iba1 immunostaining in Cingulate, Insula, primary motor (M1) area, secondary motor (M2) area, Pre-frontal, the somatosensory cortex (S1 and S2). (C) Average density values of GFAP immunostaining in Cingulate, Insula, primary motor (M1) area, secondary motor (M2) area, Pre-frontal, the somatosensory cortex (S1 and S2) (n ​= ​5). Cortex areas and hippocampus were analyzed fifteen days after sham or PSNL surgery (n ​= ​5). The vertical lines indicate the S.E.M and the symbols indicate: ∗∗P ​< ​0.01 and ∗∗∗P ​< ​0.001 denote significance levels when compared with the sham-operated group (Unpaired *t*-test).

Since alterations in glia play a significant role in neuropathic pain and mood disorders (Burke et al., 2013), we analyzed changes in microglia and astrocytes fifteen days after PSNL using immunohistochemistry for the microglial marker Iba1 and the astrocyte marker GFAP in the hippocampus, prefrontal, anterior cingulate and anterior insula, somatosensory cortex (S1 and S2), motor areas (primary (M1) and secondary (M2) motor cortex). First, we investigated the hippocampus because it is involved in mood disorders and nociception (Lee et al., 2011). Representative sections for immunohistochemistry are shown in Fig. 7 for Iba1. There was robust staining in all areas of the hippocampus, including the CA1, CA2, CA3, CA4, and DG regions with Iba1 and GFAP in sham-operated animals (Fig. 8A). After PSNL, the density of immunoreactivity for Iba1 was significantly lower than the sham-operated group (SO ​= ​0.2134 ​+ ​0.01923; PSNL ​= ​0.02165 ​+ ​0.00088 optical density; P ​< ​0.001). Similarly, hippocampal sections from the PSNL group showed significantly lower GFAP immunoreactive density when compared to the sham-operated group (SO ​= ​0.2654 ​+ ​0.02068; PSNL ​= ​0.02107 ​+ ​0.008645; P ​< ​0.001) (Fig. 8A).

To test glial changes in the cortex, fifteen days after surgery, we analyzed different regions of the limbic system (prefrontal, anterior cingulate and anterior insula), the somatosensory cortex (S1 and S2) and motor areas (primary (M1) and secondary (M2) motor cortex), all areas involved in nociception. A significant increase in Iba1 staining occurred in the pre-frontal cortex when compared to the sham group (SO ​= ​0,31 ​+ ​0,006; PSNL ​= ​0,41 ​+ ​0,016) P ​< ​0.01). There was no difference in Iba1 in the anterior cingulate, anterior insula, M1, M2, S1, and S2 cortical areas (Fig. 8B). There was also no difference in the astrocyte marker for GFAP in all mice cortical regions (prefrontal, anterior cingulate, insular, M1, M2, S1, S2) (Fig. 8C).

## 4 Discussion

Mice with partial sciatic nerve ligation present mechanical and thermal hyperalgesia, with data similar to that, observed in prior studies (Córdova et al., 2013; Meotti et al., 2006). Uniquely, we show that after sciatic nerve ligation, animals also show depressive-like and anxiolytic-like behaviors measured using the tail suspension task, elevated plus maze, and open field test. Animals also presented with short-term memory deficits. Prior studies have examined the correlation between nociceptive and mood behavior in animal models, as after nerve injury, with results that demonstrate depression and anxiety-related with chronic pain (Arora and Chopra, 2013; Blackburn-Munro and Blackburn-Munro, 2001; Rouwette et al., 2012; Moriarty et al., 2011). In parallel to behavioral changes, we observed, for the first time, changes in cytokines and growth factors in the nerve and spinal cord. We further show decreased microglia and astrocyte density in the hippocampus and increased microglial density in the prefrontal cortex. Together data suggests supraspinal glia is involved in the pathophysiology of nerve damage and related to mood disorders and cognitive impairment associated with nerve injury.

### 4.1 Neuropathic pain induces mood disorders and memory loss

Clinically, it is suggested that chronic pain might induce mood disorders like depression and anxiety (Gai et al., 2014). Chronic pain results in changes in areas involved in the affective and emotional dimension of pain in rodents – e.g., distress, pain intensity - as much or more than regions affected in the sensory components of pain processing (Blom et al., 2014; Yalcin et al., 2014). The current study shows that the induction of neuropathic pain resulted in depressive and anxiolytic behaviors in mice, thus supporting this hypothesis. Previous studies have shown that animals submitted to PSNL have neuropathic pain and anxiety and depression-like behavior (Nishinaka et al., 2015; Dimitrov et al., 2014; Kodama et al., 2011). These authors demonstrated that PSNL impairs hippocampal neurogenesis (Nishinaka et al., 2015) and that stressors, such as maternal separation, can worsen pain perception (Dimitrov et al., 2014). Although some studies have shown the relationship between chronic pain and memory disorder (Kodama et al., 2011; Rahn et al., 2013), this is the first study to show a chronic neuropathic injury that causes impairment in memory in mice. Acute activation of brain signalizing proinflammatory cytokines in response to peripheral immune activation is associated with deficits in hippocampal-dependent memory, such as aversive memory (Del Camino et al., 2010). Hippocampus modulates cognition and emotion, and hippocampal neurons are activated by noxious stimuli (Dutar et al., 1985; Fiore and Austin, 2016). Furthermore, neuroinflammation in the hippocampus is likely to influence affective behaviors by modulation of N-methyl-D-aspartate (NMDA) receptor function and/or reduction of neurogenesis by suppression of BDNF. Also, an increase in TNF-α appears to contribute to memory impairment by inhibition of long-term potentiation (LTP - persistent increase in synaptic strength following a high-frequency stimulation of a chemical synapse), via NMDA receptors (Fiore and Austin, 2016; Butler et al., 2004; Wang et al., 2013).

### 4.2 Increased cytokines and growth factors in the sciatic nerve and spinal cord after neuropathy

The current study showed a significant increase in proinflammatory cytokines (TNF-α, IL-1β, and IL6) at the site of injury and in the spinal cord, and agrees with prior studies (Clark et al., 2013; Bastien and Lacroix, 2014). Clinical evidence has shown the increase in inflammatory markers in the periphery and central nervous system of patients with depression, appear to be related to the inflammatory hypothesis of mood disorders, mainly by activating glial cells that may be secreting these proinflammatory cytokines (Dahl et al., 2014; Maes et al., 1993; Jo et al., 2015). Studies in humans demonstrate that major depressive disorder has an inflammatory component - elevated blood levels of the TNF-α and IL-6 (Liu et al., 2012) - and individuals with neuropathy associated with depression have higher levels of TNF-α than individuals with neuropathy without depression (Üçeyler et al., 2007). Proinflammatory cytokines from the periphery could be transmitted via primary afferent fibers, indirectly projecting for many supraspinal regions involved in the transmission of pain and humoral processes (Fiore and Austin, 2016). This information suggests that the symptoms of pain and depression may share the same inflammatory mechanism (Walker et al., 2014). Further, elevated spinal growth factors, especially BDNF, are pronociceptive after nerve injury (Coull et al., 2005), and low levels of they in the supraspinal areas (like hippocampus) are associated with stress, depression, and pain (Fiore and Austin, 2016). Neurotrophins are associated with neuropsychiatric diseases, such as major depressive disorder, especially for their importance in neuronal development and differentiation, as well as neuron survival (Fiore and Austin, 2019). Surprisingly, in this experimental model, we show no changes in growth factors in the hippocampus and cerebral cortex. However, the peripheral increase in growth factors (BDNF and NGF) and inflammatory cytokines initiated by nerve damage may be involved in the pathophysiology of mood disorders as they are related to pain (Fiore and Austin, 2016).

### 4.3 Microglia and astrocytes hippocampus density reduction and microglial prefrontal cortex density increases after mice neuropathy

Stressful events, like chronic pain, could activate neural circuits involved in emotional responses, cognition, and endocrine control. Some brain regions included in this process are the brainstem, the amygdala, the hippocampus, and the prefrontal cortex (Fiore and Austin, 2016; Rotheneichner et al., 2014; Fasick et al., 2015). Rats with neuropathy have behavioral changes, such as disabling a valid state, related to neuroinflammation in the PFC and hippocampus (Fiore and Austin, 2019). Also, abnormalities are observed in the hippocampus and PFC, such as reduced volume and neurogenesis, in patients with mood disorders (Fiore and Austin, 2016; Rotheneichner et al., 2014). The increase of inflammatory substances, causing neuroinflammation in the PFC after nerve injury, as seen in other brain areas, could lead to affective disturbances, including anxiety- and depressive-like behaviors as well as breaking to sleep/wake cycles (Burke et al., 2013; Fiore and Austin, 2016; Shao et al., 2015; Nascimento et al., 2015; Wang et al., 2012; Narita et al., 2011). In the current study, we observed an increase in microglial (Fiore and Austin, 2016; Shao et al., 2015) activation in PFC after nerve injury. In agreement with the current study, nerve injury expression levels of 1147 different transcripts, specially GFAP, were downregulated in the PFC of mice (Alvarado et al., 2013). Nerve injury also elevates IL-1β in the PFC, and blockade of IL-1β activity with IL-1ra reverses depressive-like behavior (Norman et al., 2010). Similarly, blockade of serotonin with fluoxetine in PFC leads to depressive-like symptoms (Nascimento et al., 2015). Together, these data suggest PFC plays a significant role in depressive-like behaviors.

In this study, we also found that regions of the cortex, such as cingulate and somatosensory area (S2), presented lower microglial density after induction of neuropathy. Indeed, imaging studies in humans with complex regional pain syndrome identified specific relevant brain regions, which include somatosensory cortex, cingulate, and thalamus (Bentley et al., 2016; Pahapill and Zhang, 2014).

In the present study, we observed a dramatic decrease in the optical density of hippocampus microglia and astrocyte markers in all animals with nerve injury. In agreement, prior studies show a reduction in the number and volume of astroglial cells after stress (Czéh et al., 2006). Some studies show nerve injury induces a proinflammatory response in the PFC and hippocampus, with upregulation of proinflammatory cytokines, TNF-α, IL-1β, and IL-6 (Fiore and Austin, 2016; Fasick et al., 2015). Glial-cytokine-neuronal adaptations in the hippocampus of animals with neuropathy may lead to functional recovery and coping, however, contribute to the emergence of debilitating and ongoing changes in affective state (Fasick et al., 2015). The current study extended these findings by showing an increase in microglia activation of the PFC, but no changes in the neurotrophins NGF and BDNF. Interactions between the PFC and the hippocampus have been well-characterized in rats and monkeys (Öngür and Price, 2000; Fiore and Austin, 2019). Further studies of these structures could be vital for understanding several highly debilitating and prevalent psychiatric disorders, especially since these circuits are readily plastic and may be modified in the usual domain of human experience (Kolling et al., 2012; Fiore and Austin, 2019).

In summary, we reported pathological pain induced by PSNL interferes with multiple tasks such as cognition and emotional states. Specifically, we show PSNL causes depressive-like and anxiety-like behaviors as well as impairment in short-term memory in mice. Our data indicate the possible involvement of supraspinal astrocytes and microglia, cytokines, and growth factors, in the development of mood disorders and memory loss in animals with neuropathy. The present work suggests that the injury itself drives the changes in mood and cognitive function, and these results may help to understand the increase in the prevalence of mood disorders and cognitive deficits in chronic pain conditions. Knowledge of CNS plastic changes in neuronal and glial cells, as well as the substances released by them, may enable the development of new therapies to improve the quality of life of patients suffering from painful conditions. Futures studies should take the exam if pain treatment alters mood and memory and other mechanisms involved in these conditions.

## Declaration of competing interest

The authors declare the following financial interests/personal relationships which may be considered as potential competing interests: Morgana D. da SilvaGiselle GuginskiKarina L. SatoLuciana Sayuri SanadaAdair R. S. SantosKathleen A. Sluka.
